# Soft Matrices Suppress Cooperative Behaviors among Receptor-Ligand Bonds in Cell Adhesion

**DOI:** 10.1371/journal.pone.0012342

**Published:** 2010-08-23

**Authors:** Jin Qian, Huajian Gao

**Affiliations:** School of Engineering, Brown University, Providence, Rhode Island, United States of America; Massachusetts Institute of Technology, United States of America

## Abstract

The fact that biological tissues are stable over prolonged periods of time while individual receptor-ligand bonds only have limited lifetime underscores the critical importance of cooperative behaviors of multiple molecular bonds, in particular the competition between the rate of rupture of closed bonds (death rate) and the rate of rebinding of open bonds (birth rate) in a bond cluster. We have recently shown that soft matrices can greatly increase the death rate in a bond cluster by inducing severe stress concentration near the adhesion edges. In the present paper, we report a more striking effect that, irrespective of stress concentration, soft matrices also suppress the birth rate in a bond cluster by increasing the local separation distance between open bonds. This is shown by theoretical analysis as well as Monte Carlo simulations based on a stochastic-elasticity model in which stochastic descriptions of molecular bonds and elastic descriptions of interfacial force/separation are unified in a single modeling framework. Our findings not only are important for understanding the role of elastic matrices in cell adhesion, but also have general implications on adhesion between soft materials.

## Introduction

Understanding how cells sense their mechanical environment has become a topic of central importance in cell biomechanics [1], [2]. Recent progress in the design and application of artificial cellular substrates mimicking the extracellular matrix (ECM) has revealed the extraordinary ability of cells to adjust their shape, adhesion, motility and intracellular organization to physical and chemical changes in their immediate surroundings [3], [4]. One of the most impressive advances in this area is the realization that cell adhesion depends sensitively on the rigidity of the extracellular environment [5]–[9]. Cells cultured on rigid glass or plastic dishes typically develop discrete micron-sized adhesion sites, commonly referred to as focal adhesions (FAs), in which cytoskeletal actin bundles are anchored on substrates via dense clusters of receptor-ligand bonds [5]. It has been shown that focal adhesions decrease in size for cells cultured on increasingly softer substrates with elastic modulus varying in the physiological range of 1–100 kPa [6], that cells cultured on elastically nonhomogeneous substrates tend to actively migrate towards the stiffer regions [7], [8], a phenomena know as durotaxis, and that the fate of mesenchymal stem cells can be controlled by matrix stiffness [9].

Cells adhere specifically to ECM via focal adhesions, where receptors on cell membrane form multiple bonds with ligands such as the ECM protein fibronectin on the extracellular side [10], while connecting with the actin cytoskeleton via a cytoplasmic adhesion plaque composed of many different proteins on the intracellular side [11]. The number of receptor-ligand bonds in such multiple-bond adhesion can range from just a few in short-lived focal complexes to as many as 10^5^ in relatively stable focal adhesions. Serving as the sole anchorage between cell and ECM, these bond clusters are usually exposed to forces induced by external physical interactions such as blood flow, as well as those generated by cell's own contractile machinery as stress fibers made of bundles of actin filaments and myosin II motors actively pull FAs towards the inside of the cell. The growth or shrinkage of an FA is strongly dependent on the forces applied on it. Focal adhesions tend to elongate in the cell-substrate interfacial plane with long axis aligned in the force direction [12]. Inhibition of the contractile stress leads to dissolution of cytoskeleton and disappearance of FAs [13]. When myosin II activity is suppressed, application of an external force, irrespective of its physical origin, is found to stimulate growth of FAs in the direction of the force [14]. In the case of cell-generated tension, the size of mature FAs can reversibly increase or decrease in response to the magnitude of cellular tension, with force per unit area (stress) maintained near a constant value around 5.5 kPa which is remarkably similar among different cell types [15], [16].

During the past two decades, tremendous progress has also been made on quantitative characterizations of the behavior of molecular bonds under force, mainly on the level of single molecules or bond clusters between rigid media. Unlike adhesive interactions at macroscale, individual receptor-ligand bonds will dissociate sooner or later with or without an applied force. Intensive studies, including experiments based on dynamic force spectroscopy [17]–[19] and theoretical models [20], [21], have been carried out to understand the behavior of single molecular bonds under an applied force. The process of bond dissociation is often regarded as thermally assisted escape over a potential energy barrier [22], [23]. Application of an external force changes the energy landscape and therefore influences the rupture process. For time-independent loading, both theories and experiments have indicated that the dissociation rate 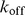 of a closed bond increases exponentially with a force 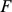 acting on the bond as [24]

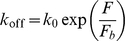
(1)where 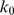 is the spontaneous dissociation rate in the absence of the force and 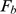 is a force scale typically in the pN range [23].

**Figure 1:**
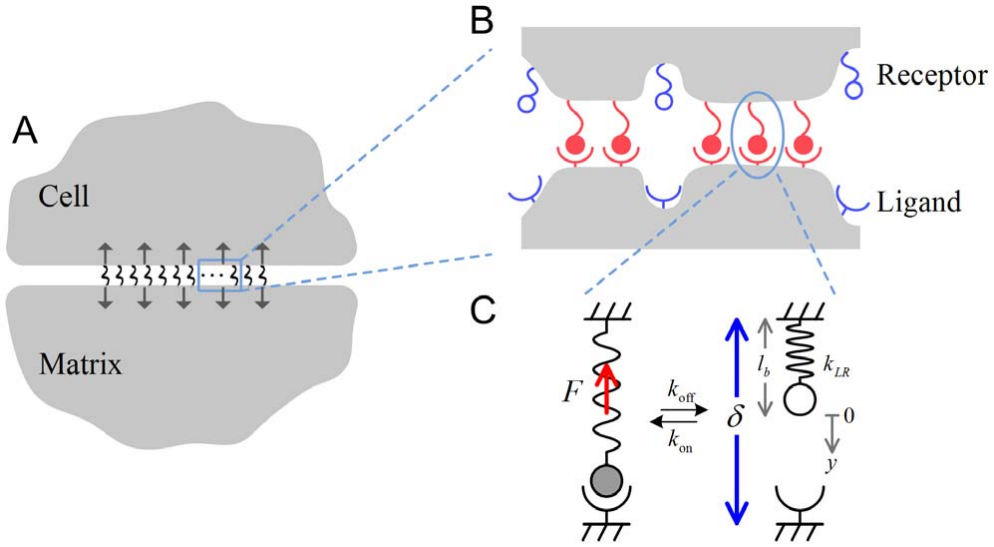
A stochastic-elastic model of focal contact demonstrating the effect of cell/matrix compliance. (A) A single adhesion patch between two elastic media (cell and extracellular matrix) subjected to a uniform tensile stress directly applied along the interface. In this case, the applied load is nominally equally shared among all bonds, independent of the system elasticity. (B) The elastic recoil at open bonds increasing the surface separation at these bond locations and suppressing receptor/ligand rebinding that is necessary for stable adhesion. (C) Bond transition between closed and open states at force-dependent dissociation and separation-dependent association rates.

For failure of a multiple-bond adhesion, one must take into account the fact that individual bonds can rebind after they break, until the whole adhesion is detached. The analysis of Evans & Ritchie [20] did not consider such rebinding, but theoretical considerations by Seifert [25] indicated that bond rebinding can greatly enhance the adhesion lifetime. In a cluster made of parallel bonds, a specific pair of bond can break and reform multiple times as long as there exist unbroken cross-bridges between the surfaces. For a ligand on a substrate surface and a receptor tethered to a cell wall by a linear spring with stiffness 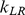 and rest length 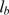, the binding or rebinding rate 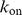 can be assumed to depend on the cell-substrate surface separation 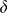 as [26]–[29]

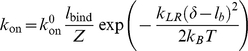
(2)where 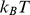 is the thermal energy (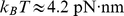 at physiological temperature), 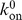 is a reference association rate when the receptor-ligand pair are within a binding radius 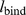, and 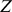 is the partition function for the receptor confined in a harmonic potential between 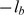 and 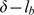 (Fig. 1C) [28].

Bell pioneered a thermodynamic framework of cell adhesion [24]. Subsequently, the process of adhesion or deadhesion of cells from substrates was modeled via peeling tests that are familiar in engineering design but is made more complicated by the biological interface and geometry involved [30], [31]. More recent progresses have been made in modeling curved biological membranes spreading on a flat substrate mediated by binder diffusion [32], [33], as well as receptor-mediated cellular uptake and release of viruses or nanoparticles [34]. Erdmann and Schwarz [35], [36] studied the stochastic effects of a cluster of uniformly stressed molecular bonds transiting between open and closed states under the influence of thermal fluctuation. Based on the solutions to a one-step master equation, Erdmann and Schwarz demonstrated that clusters below a critical size behave like a single molecular bond with a finite lifetime while those above the critical size survive over a much prolonged lifetime due to the cooperative effect of clustering. Therefore, adhesion size can play a very important role in the stability of a bond cluster: small clusters can easily switch between adhesion and deadhesion, as in short-lived focal complexes, while large clusters tend to have a much longer lifetime similar to stable focal adhesions. Qian et al. [28], [29] extended the work of Erdmann & Schwarz to including the effects of cell/matrix elasticity and non-uniform stress distribution on the stability of a single or a periodic array of adhesion clusters under normal and inclined loads, with results showing a size-dependent transition between uniform and crack-like distributions of interfacial traction, a window of cluster size for relatively stable adhesion and an optimal size for maximum adhesion strength. Analysis by Lin and Freund [37] based on a direct analogy between focal adhesions and periodic cracks led to similar conclusions.

In spite of the tremendous progresses in experimental and theoretical studies of cell adhesion over several decades, precisely how cells sense and respond to matrix stiffness is still an open question. Chan and Odde [38] investigated stiffness sensing by constructing a stochastic model of the “motor-clutch” force transmission system, where molecular clutches link F-actin to the substrate and mechanically resist myosin-driven F-actin retrograde flow. Their model predicts two distinct regimes in retrograde flow and integrin traction forces for stiff and soft substrates. Walcotta and Sun [39] proposed a model to show that the stiffness of the substrate directly influences differential formation of stress fibers in cytoskeleton and ultimately leads to changes in intracellular biochemistry. We have previously discussed the lifetime and stability of molecular bond clusters under soft-matrix-induced stress concentration at the cluster edges [28], [29]. In this paper, we will show a more striking effect that soft matrices can suppress the cooperative behaviors in a multiple-bond adhesion with or without stress concentration at the adhesion edges. The essence of this effect is that the local elastic recoil following a bond rupture event can lead to large surface separation, thereby preventing future rebinding of the bond. In the following, we will show this effect via a coupled stochastic-elastic modeling framework similar to our previous work [28], [29].

## Results

### Model

To understand how clusters of molecular bonds work together to sense and respond to the stiffness of their local environment, here we construct an elastic-stochastic model as follows. Cell and ECM surfaces are separated from each other under an applied load while receptors and ligands form transient attachments and undergo stochastic rupture and rebinding according to the rate equations in Eqs. (1) and (2) (Fig. 1A). Only specific adhesion via opposing receptor-ligand pairs is considered and secondary non-specific interactions are ignored. One side of the adhesion is an elastic medium mimicking the adhesion plague on the cytoplasmic side of a cell and the other side represents an elastic substrate (ECM). The Young's modulus and Poisson's ratio are 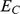, 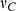 for the cell and 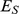, 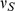 for the substrate. It will be convenient to define a reduced elastic modulus 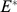 according to the convention of contact mechanics [40]:
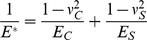
(3)Within the adhesion domain, a number of molecular bonds are fixed at spacing 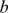, corresponding to a bond density of 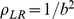. A slice of the system with out-of-plane thickness 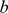 is considered in a plane strain model. In this set-up, the total number of molecular bonds in a cluster of size 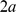 is 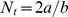.

Each bond is modeled as a Hookean spring with stiffness 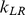 and rest length 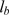. Suppose that the bond cluster between the two dissimilar elastic media is subjected to a uniform tensile stress 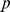 (Fig. 1A). Instead of a remotely applied force which tends to induce a non-uniform distribution of bond forces within the cluster [28], this uniformly applied stress 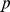 at the cell-substrate interface ensures that all bonds are nominally subjected to an equal force. In this setting, the effects of a nominal stress concentration within the cluster are excluded from the analysis, but we will show that the cell/substrate compliance can still strongly influence the adhesion lifetime because soft media substantially diminish the separation-dependent rebinding rate as a result of the elastic recoil at open bonds (Fig. 1B).

Assume that the applied stress 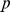 causes a nominal force 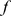 (normalized by the force scale 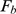 in Eq. (1)) acting on individual bonds. When both cell and substrate are relatively stiff compared to the molecular bonds, our model is reduced to the case of a total pulling force 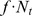 acting on a molecular cluster between two rigid bodies. Suppose that 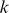 (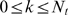) bonds are closed and 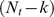 bonds are open at a given time 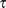 (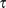: real time normalized by the time scale 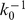 in Eq. (1)). The 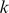 closed bonds would share the total applied force equally, so that the actual force acting on each closed bond is 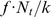. Each of the 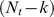 open bonds is assumed to rebind at a separation-dependent rate described in Eq. (2). For the initial condition 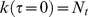, the average lifetime of the molecular bond cluster, 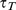, defined as the mean first passage time reaching the failure state 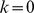, can be calculated analytically as [41]

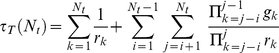
(4)under the reflecting boundary condition at 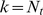 and absorbing boundary condition at 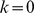. The above equation accounts for all possible pathways transiting from the initial cluster size 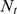 towards the absorbing boundary 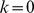 with their statistical weights. Under the present setting of molecular clusters between rigid media,
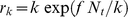
(5)

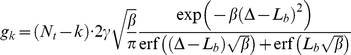
(6)where 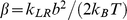 and 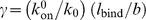 is a prefactor for bond rebinding; 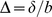 and 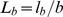 are the normalized surface separation and bond rest length, respectively. The surface separation 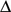 in Eq. (6) is also a function of 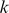 as 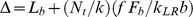.

If 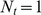, Eq. (4) is reduced to 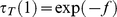, which is just the lifetime of a single molecular bond. In the case of zero rebinding, the second term of Eq. (4) vanishes and the cluster lifetime becomes 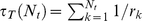, which, in the absence of an applied force, is further reduced to 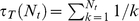, corresponding to the 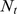-th harmonic number.

### Stochastic-elasticity coupling

However, in the presence of elastic deformation (due to the compliance of either cell or ECM), the dissociation and association rates in Eqs. (1) and (2) would also depend on the local force and surface separation at a bond location within the adhesion domain. The strongly decaying behavior of rebinding rate with increasing separation, as given in Eq. (2), is expected to play a very important role in the stability of molecular bond clusters when the elasticity of the system is considered. Once the opposing surfaces are separated locally at open bonds by more than a critical distance, bond rebinding becomes hardly possible and the cluster is expected to undergo a catastrophic failure process.

The analytical solution to the original master equation is no longer available in the case of compliance-induced spatially dependent rupture and rebinding rates. The elastic descriptions of interfacial force/surface separation between cell and substrate can be incorporated into the stochastic dynamics of bond clusters through an elastic Green's function approach (Methods: Elasticity modeling). A Monte Carlo scheme has been developed based on Gillespie's algorithm [42], [43] to numerically solve the spatio-temporal process governed by the master equation. The basic idea is to cast stochastic trajectories of cluster evolution in accordance with the above described reaction rates and then average over many independent trials to obtain useful statistical information. In our Monte Carlo simulations, each bond location 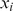 is considered an independent reaction site where the next event will be bond rupture at rate 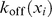 if the bond is currently closed, and bond rebinding at rate 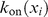 if the bond is currently open. The reaction rates, 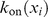 and 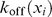, are determined from the computed forces on closed bonds and surface separations at open bonds. The “first reaction method” of Gillespie's algorithm [42], [43] is used to determine when and where the next reaction will occur through random number generation (Methods: Monte Carlo simulation). When the binding state of any bond (open versus closed) has undergone a change, an update of the force and surface separation at all bonds is performed using the associated elastic Green's function, and the results are used to determine the subsequent events. This coupling between elasticity modeling of interfacial traction/separation and stochastic events starts at the initial state when all bonds are closed and the process proceeds until all bonds within the adhesion domain become open. The total elapsed time 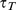 (real time normalized by 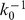) is recorded as the adhesion lifetime. The statistical lifetime is obtained from an average of 1,000 independent simulation trajectories for each given parameter set. For relevant physical/biological parameters used in the simulation, we adopt the following typical values: 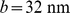, 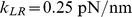, 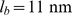, 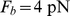, 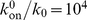 and 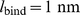 unless stated otherwise.

### Analysis

The elastic recoil of a broken bond pair can lead to large local surface separation, thereby preventing future rebinding of the bond and killing the effects of bond cooperation. To demonstrate that this effect exists independent of stress concentration, we consider a single adhesion patch subjected to a uniform stress 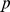 applied directly along the interface over the adhesion domain 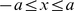 between cell and substrate. The governing equation under plane strain conditions is [40]

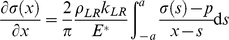
(7)where 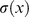 is the traction within the adhesion domain and 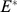 has been defined in Eq. (3). The solution to Eq. (7) is simply 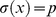 when all of the bonds are closed. We see that, instead of a remotely applied force which tends to induce a non-uniform distribution of bond forces with concentrated forces at edges within the cluster [28], the uniformly applied stress 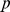 at the interface ensures that all bonds are nominally subjected to an equal force. In this setting, the effects of cell/substrate stiffness on the adhesion lifetime are not due to a nominal stress distribution.

In a cluster of molecular bonds at the cell-substrate interface, breaking one bond bears some resemblance to a finite crack of size 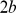 (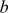 is the bond spacing) in an infinite elastic media. A rough estimate of the elastic recoil at the center of the crack is [44]

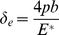
(8)The tensile stress 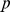 also induces an average separation between the cell and substrate, i.e.
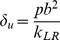
(9)where 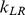 is the bond stiffness. The relative contributions of the elastic recoil 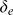 and the average separation 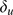 at an open bond are then measured by the parameter
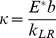
(10)In the limit of 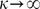, the cell and substrate are relatively rigid compared to the molecular bonds, and the cell-substrate separation is almost uniform along the interface. In this limit, the bonds behave as a cluster between rigid bodies discussed in Eq. (4). In the opposite limit of 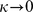, the cell and substrate are relatively soft with respect to the molecular bonds, and the local elastic recoil dominates over the averaged cell-substrate separation.

### Simulation results

**Figure 2:**
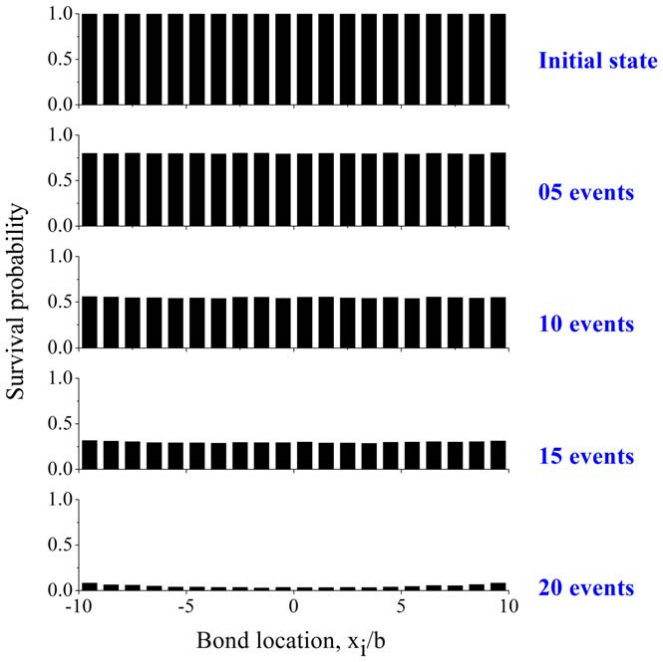
Failure mode of molecular bond clusters subjected to nominally uniform stress distribution. Averaged bond survival probability versus bond location 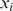 (normalized by bond spacing 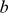) for 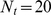, 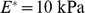 and 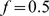. The snapshots indicate that there is no stress concentration in the adhesion domain and the molecular cluster fails in a uniform mode independent of bond location. The fact that 20 events almost break the cluster of 20 bonds suggests that bond rebinding has been rare during the failure process.

To verify that the effects of non-uniform stress distribution are indeed excluded in the present study, we first simulated a focal adhesion cluster consisting of 20 bonds. The reduced modulus of cell and substrate is taken to be 10 kPa and the force per bond (normalized by 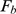) is fixed at 0.5. Fig. 2 plots the survival probability versus bond location by averaging the cluster state over 10,000 independent trajectories during cluster evolution. We see that the failure mode of the adhesion is uniform, similar to the equal-load-sharing case investigated by Erdmann and Schwarz [35], [36]. The fact that merely ∼20 events break the cluster of 20 bonds suggests that bond rebinding has been rare during the failure process.

**Figure 3:**
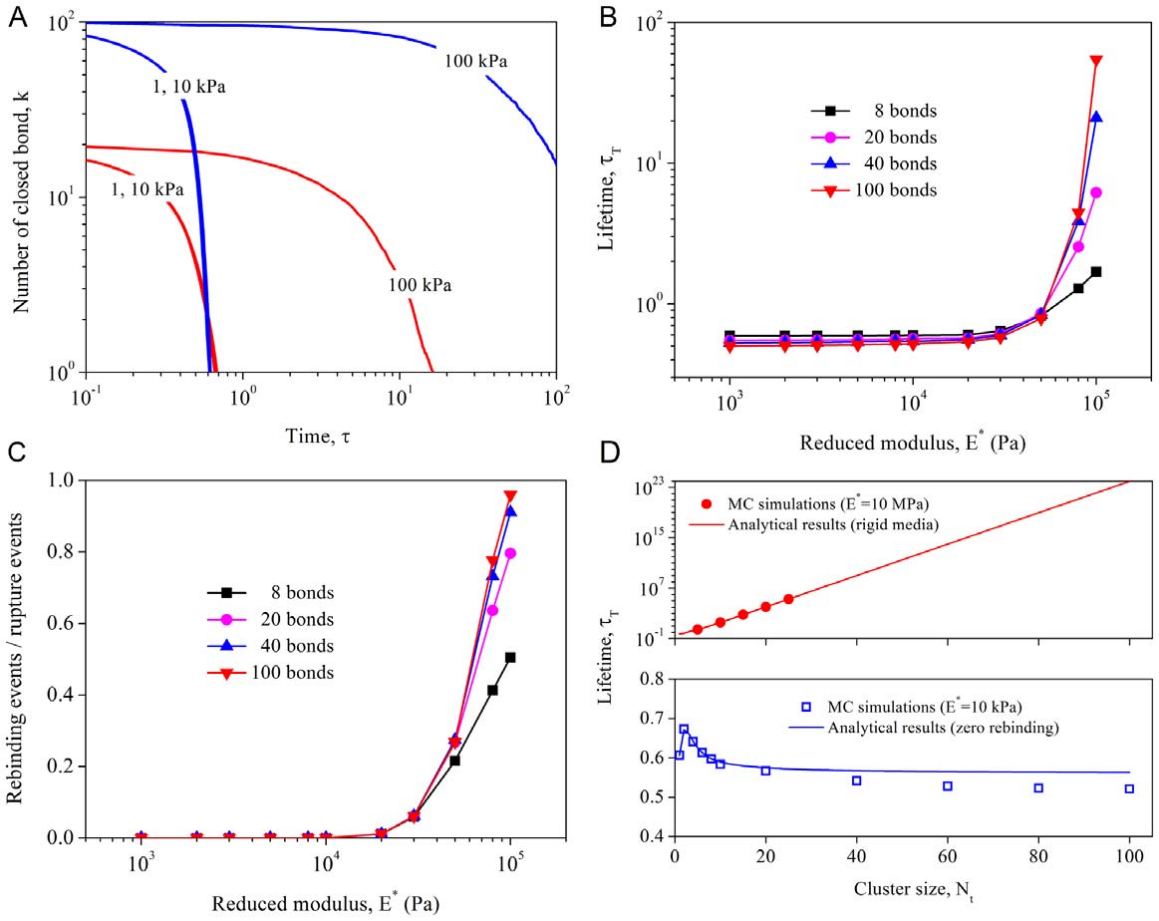
Effect of cell/substrate stiffness on bond rebinding and adhesion lifetime. (A) The number of closed bonds 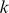 as a function of time 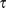 by averaging 1,000 Monte Carlo trajectories for different values of the reduced modulus 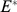 of the cell and substrate (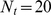 and 100). The load level 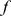 is fixed at 0.5. (B) The cluster lifetime 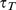 as a function of the reduced modulus 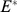 for different cluster sizes (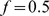). (C) The ratio of total events between bond rebinding and bond rupture influenced by the reduced modulus 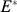 (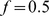). (D) Analytic results of the cluster lifetime 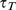 for the cases of rigid media (Upper) and zero rebinding (Lower), compared to the Monte Carlo simulations for stiff (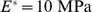) and soft (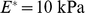) cell and substrate (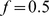).

Cell/substrate stiffening can enhance bond rebinding and stabilize molecular clusters by decreasing the local elastic recoil at open bond locations. Fig. 3A plots the number of closed bonds 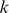 as a function of time 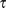 by averaging Monte Carlo trajectories for different values of the reduced modulus 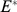. Two cluster sizes, 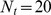 and 100, are considered and the load level 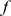 is fixed at 0.5. All clusters fail after a period of time but those between stiffer cell/substrate can sustain much longer lifetime. The cluster lifetime 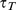 as a function of the reduced elastic modulus 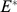 for different values of the cluster size 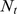 is shown in Fig. 3B. For cell/ECM with physiological 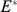 value within 1–100 kPa, the cluster lifetime 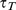 is reduced by two orders of magnitude from stiff to soft cases. By comparing the results with those of clusters subjected to a remote tensile stress [28], we find that the molecular clusters generally have longer lifetime in the absence of pre-existing stress concentration. For given parameters 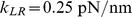 and 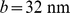, we calculate that 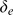 and 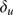 are actually comparable when 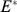 is around 10 kPa. In this case, the local elastic recoil in addition to the average interface separation causes large reductions in cluster lifetime by decreasing the probability of bond rebinding. This is confirmed by tracking the ratio of total events between bond rebinding and bond rupture during the cluster evolution, as indicated in Fig. 3C.

At a fixed cluster size, focal adhesions become more and more stable as cell and ECM stiffen, approaching the behavior of clusters between two rigid bodies, given by Eq. (4). For very soft cell/ECM, the surface separation at open bond locations is so large that rebinding becomes hardly possible. Removal of all rebinding terms in Eq. (4) gives
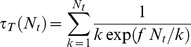
(11)under the condition of equal load sharing. This result can serve as an estimate of lifetime for a molecular cluster between very soft cell and substrate. As shown in Fig. 3D, the Monte Carlo simulations of the cluster lifetime 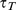 for stiff (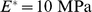) and soft (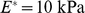) cell/substrate agree well with the analytical predictions for the cases of rigid media and zero rebinding (Eqs. (4) and (11)). Therefore, the way that cytoskeleton/ECM stiffness influences FA stability does not rely solely on how the load is transmitted in the adhesion region. Even for molecular clusters under initially uniform pulling forces, the cell/matrix compliance can still destabilize focal adhesions by suppressing rebinding of open bonds.

**Figure 4:**
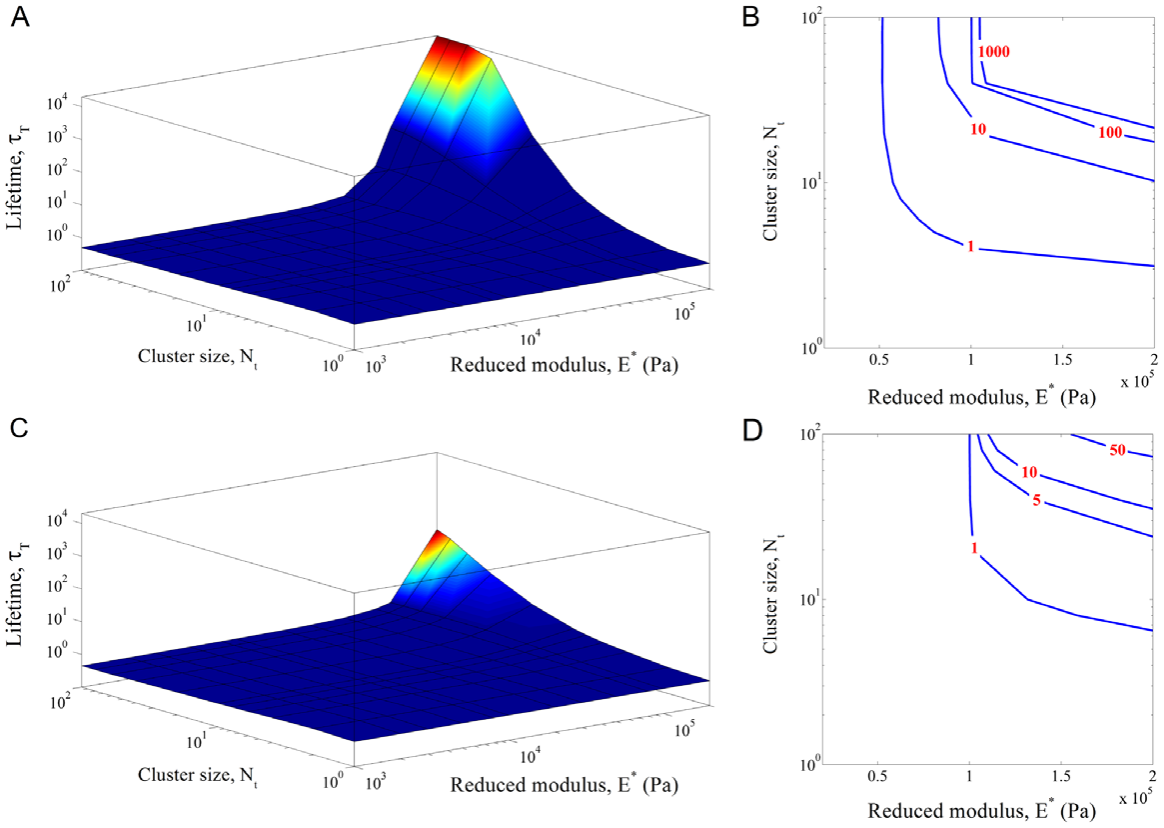
The cluster lifetime over different values of the reduced modulus and cluster size. (A, B) 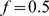; (C, D) 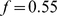. The surface (A, C) and contour (B, D) plots show that either small cluster size or soft cell/substrate leads to unstable adhesion of molecule bond clusters. In (A), the 4 data points with longest lifetime are subjected to a simulation cutoff 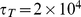.

We further perform simulations on the cluster lifetime 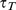 as a function of cell/substrate stiffness 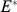 and cluster size 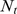 by imposing different levels of load on the adhesion patch. The two-dimensional surface and contour plots of cluster lifetime in Fig. 4 show that reducing load generally stabilizes the cluster and leads to longer lifetime. The cluster lifetime can increase by many orders of magnitude via cell/substrate stiffening (Fig. 4 A, C). In a map of cell/substrate stiffness and cluster size, having either small cluster size or low cell/substrate stiffness ends up with unstable adhesion, and prolonged lifetime is only possible for clusters with sufficiently large size and high stiffness (Fig. 4 B, D). Small clusters resemble single-molecule-like behavior due to statistic effects while very compliant cell/substrate leads to single-molecule-like adhesion by killing the rebinding events (birth rates).

**Figure 5:**
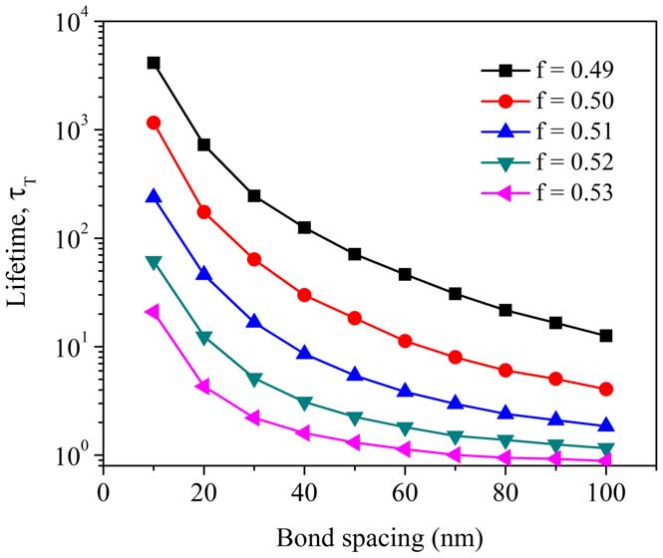
Effect of bond spacing on bond cooperation/rebinding and adhesion lifetime. The cluster lifetime 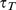 is plotted as a function of the bond spacing for different levels of the applied load 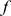 (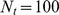, 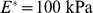).

Guided by the scaling law in Eq. (10), the cooperation of molecular bonds in focal adhesions is also influenced by the spacing between neighboring bonds. Experiments have revealed that focal adhesion is inhibited and cells do not spread for ligand spacing larger than 72 nm while formation of focal contacts and cell spreading to a pancake-like shape can operate normally only for ligand spacing smaller than 58 nm [45]. In Fig. 5, we investigate the cluster lifetime 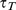 by varying the actually bond spacing, 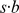, through a numerical factor *s* for different levels of load on the adhesion cluster. Indeed, increasing bond spacing lowers the cluster lifetime by orders of magnitude due to decreased bond cooperation, depending on the magnitude of the applied load. This is qualitatively consistent with the experimental observations [45].

## Discussion

Fig. 4 shows that increasing load level tends to destabilize clusters of molecular bonds. How to compare this result to the experimental observations by Riveline and coworkers [15] that more force stimulates the growth of focal adhesions? This can be interpreted following our present study as cells attempting to control the FA dynamics through actively tuning the effective Young's modulus 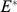 of the adhesion system by means of stress fiber stiffening. When mechanical force is applied, actin filaments in cytoskeleton will be lengthened and subject to isotropic-to-nematic transition. The significance of the formation of actin stress fibers is that cells can locally stiffen the part of cytoskeleton that is connected to an FA through contractile forces induced by Myosin II activities. Moreover, the elastic modulus of cytoskeleton can change over several orders of magnitude in response to different levels of myosin-II-driven contractility [46]–[48]. On the effect of cytoskeleton stiffness, our model shows that increasing stress on actin stress fibers associated with an FA induces local stiffening of the cytoskeleton and tends to enhance bond rebinding and stabilize FAs. Therefore, a sufficiently large cytoskeletal stress is not only beneficial but also necessary to maintain stable adhesion. However, once cytoskeleton is stiffened, further increasing force would destabilize adhesion.

The effects of the reduced elastic modulus 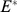 (Eq. (3)) of cell and matrix on bond rebinding and adhesion lifetime, independent of how the load is distributed within focal adhesions, imply that very soft substrates tend to diminish the adaptive capability of cells by suppressing bond rebinding irrespective of the cytoskeleton stiffness, which can prevent short-lived focal complexes from maturing into stable focal adhesions. This is also in qualitative agreement with the experimental observations that stable and large FAs can only form on sufficiently rigid substrates [5], [6]. The fact that FAs on stiff substrates are more stable provides a possible driving force for cells to migrate towards stiffer part of the substrate [7], [8]. On hard substrates, the reduced elastic modulus 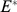 tends to be dominated by the stiffness of the cytoskeleton. The cytoskeletal contractile forces can stiffen cytoskeleton by decreasing entropic elasticity of the actin network [46]–[48] and therefore benefit the long term stability of FAs. This is consistent with the experimental observations that cytoskeletal contractile forces are necessary to stabilize cell adhesion [13].

Low stiffness, which could result from the presence of a soft matrix or dissolution of cytoskeleton, has two devastating effects on focal contacts. First, it can induce severe stress concentration near the adhesion edges and crack-like failure around the rims of focal contacts, as demonstrated in our previous studies [28], [29]. Second, we have shown in this paper a more striking result that low stiffness of cell/matrix tends to increase local surface separation at open bonds and make them difficult to rebind, effectively killing the birth rate in a bond cluster. The present study indicates that the effect of elasticity in controlling bond rebinding is intrinsic in molecular adhesion between soft materials.

In conclusion, we have performed theoretical analysis and Monte Carlo simulations to demonstrate that the cell/substrate compliance plays an essential role in controlling focal adhesion stability even for molecular clusters under nominally uniform pulling forces. This effect arises from the stiffness-dependent elastic recoil at cell-substrate interface and separation-dependent rebinding of molecular bonds, and the way that cytoskeleton/ECM stiffness influences FA stability does not rely solely on how the load is transmitted in the adhesion region. While the effect of stress concentration in adhesive contact is well known in contact mechanics theory [49] as well as applications such as gecko adhesion [50]–[52], the role of elasticity in suppressing bond rebinding is a unique feature of molecular adhesion, and the sensitivity of focal adhesions to cell/substrate stiffness cannot be alleviated simply by removing stress concentration from the system. Generally, stiff substrate, cytoskeleton stiffening and bond cooperation through clustering are factors that contribute to the stability of focal adhesions. The modeling framework in this study that couples stochastic descriptions of molecular bonds and elastic descriptions of interfacial deformation provides a quantitative theoretical basis for the spatio-temporal processes of molecular bonds and should be generally applicable to more complex situations such as leukocytes rolling and tethering on vessel walls [53], [54] and immunological synapse formation in cell-cell adhesion [55], [56].

## Methods

### Elasticity modeling

Consider a single adhesion cluster under a uniform tensile stress 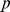 over the adhesion domain 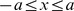. The discontinuity of the normal displacement on cell and substrate surfaces, denoted as 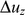, at a bond location 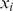 due to all the forces sustained by the closed bonds is [40]

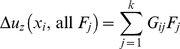
(A1)where 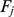 is the bond force at 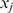
and
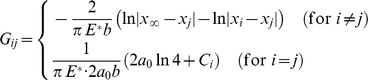
(A2)is the 2D Green's function for a concentrated force acting on an elastic half-space [40], and 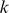 is the current number of closed bonds within the adhesion domain; 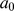 is the half-width of molecular bonds which has a typical value of 5 nm [45]; 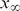 is an arbitrary reference point that does not influence the solution and 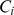 is to satisfy the condition that 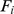 causes zero displacement at 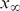. On the other hand, the tensile stress 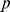 applied at the cell-substrate interface also causes displacement discontinuity at 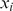, which is given by [40]

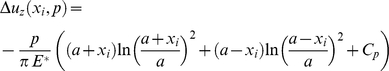
(A3)where 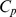 is to satisfy the condition that 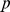 causes zero displacement at the same reference point 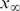.

The conditions of interface compatibility and global force balance are
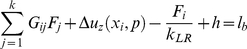
(A4)

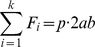
(A5)Here 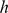 is the unknown cell-substrate surface separation in the absence of elastic deformation. Once the 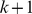 unknowns 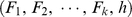 are solved from Eqs. (A4) and (A5), the surface separation 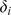 between the two elastic media can be calculated by
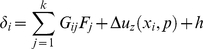
(A6)for any open bond location 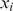.

The bond forces on closed bonds and surface separations at open bonds are used to compute the reaction rates (rupture or rebinding) of a bond cluster for any instantaneous bond configuration during the cluster evolution.

### Monte Carlo simulation

At any step during the cluster evolution, random numbers are generated to determine whether the next activity is bond rupture or rebinding, and how long it takes for the next reaction to occur. For our elasticity modeling with spatial degrees of freedom, it is also necessary to determine where the next event should occur. In the simulations, we have applied the so-called “first-reaction method” [42], [43], which was also adopted by Erdmann and Schwarz [35], [36] in their simulations under the assumption of equal load sharing.

For any simulation step of a molecular bond cluster, we need to determine a series of reaction rates denoted as 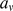, 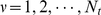 referring to a bond location, from the computed dissociation or association rates depending on the current cluster state. We generate a series of independent random numbers 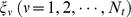, which are uniformly distributed over the interval [0, 1], and calculate the reaction time for individual reaction sites according to
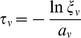
(B1)The time for the next reaction is chosen to be the smallest among 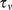, i.e.
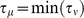
(B2)At the same time, the location for the next reaction is identified to be the site 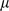 where 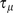 is chosen. The event type for the next reaction is “rupture” if the bond at site 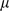 is currently closed and “rebinding” if it is currently open.

Any change of bond state requires an update of bond force and surface separation in the elasticity modeling, which are then used to determine the subsequent reaction rates 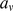.
